# Enzalutamide induced non-ischemic cardiomyopathy. A case report and review of literature on anti-androgen therapy-related cardiovascular events

**DOI:** 10.1186/s40959-023-00160-7

**Published:** 2023-01-31

**Authors:** Aswini Kumar, Aswanth Reddy, Arjun Sekar

**Affiliations:** 1 Department of Cardiology, Mercy Clinic, 7001 Rogers Ave, Fort Smith, AR 72903 USA; 2 Department of Hematology-Oncology, Mercy Clinic, 7001 Rogers Ave, Fort Smith, AR 72903 USA; 3 Department of Nephrology, RGH Center for Kidney Disease and Hypertension, Rochester Regional Health, 370 Ridge Rd E, Ste 20, Rochester, NY 14621 USA

**Keywords:** Enzalutamide, Prostate cancer, Cardiomyopathy, Hypertension

## Abstract

Prostate cancer has a very high prevalence among elder men, and this could potentially increase as longevity in many parts of the world is increasing. Early stages of prostate cancer can have surgical options, but the more advanced stages require some form of anti-androgen therapy. There are novel anti-androgen agents that were recently approved. Cardiovascular toxicity has been reported with some of these drugs. This is a novel report of likely cardiovascular toxicity due to Enzalutamide, which typically has a safer cardiovascular profile than Abiraterone.

We describe a 72-year-old male with repeated recurrence of prostate cancer with metastasis. The second time it recurred was within 2 years of the 1st recurrence and was treated with Enzalutamide.

However, within 2 weeks he developed systolic congestive heart failure that improved with stopping the drug and medical optimization.

Literature review shows that Abiraterone has more cardiovascular side effects than Enzalutamide which more commonly causes hypertension. The timeline in our case suggests Enzalutamide causing congestive heart failure which is a novel finding. This finding warrants further research regarding the safety profile of novel anti-androgen therapy. This includes risk stratification for potential cardiovascular adverse events and risk/benefit analysis prior to initiating therapy. Data on cumulative dose accumulation and risks can also be an area of future research.

## Background

Prostate cancer is the most common cancer in men in the United States [1], with an average age at diagnosis of 66 and more likely to develop in older and non-Hispanic Black men [2]. Androgen deprivation therapy (ADT) is the primary treatment modality for locally advanced and metastatic prostate cancer. Cardiovascular risk and mortality incidence are higher in patients treated with Gonadotropin releasing hormone (GnRH) analogs. Adding novel agents such as Enzalutamide and Abiraterone to the treatment regimen further increases cardiovascular risk. Enzalutamide and Abiraterone are both FDA-approved for use in metastatic castrate-resistant prostate cancer. We report an elderly patient who tolerated Abiraterone well but had significant cardiovascular morbidity following treatment with enzalutamide.

## Case presentation

Our patient is an 82-year-old Caucasian man who had non-metastatic prostate cancer in 2007 and was treated with radiation to the prostate and androgen deprivation therapy. In January 2019, he had a detectable serum prostate-specific antigen (PSA) in surveillance blood work. Imaging studies showed an anterior right seventh rib lesion and retroperitoneal lymphadenopathy. Treatment was initiated with Abiraterone and GnRH agonist, and his PSA steadily improved. Nevertheless, in September 2021, his PSA started rising. Computerized tomography showed persistent retroperitoneal lymphadenopathy, and a bone scan revealed a single new abnormal area in the left ilium. Treatment was switched to Enzalutamide from Abiraterone by the Oncologist.

Two weeks after starting enzalutamide, he presented to the emergency room with acute onset shortness of breath. An echocardiogram demonstrated new cardiomyopathy, reduced left ventricular (LV) systolic function with an ejection fraction of 20%, and severe diffuse hypokinesis. Left heart catheterization showed moderate atherosclerosis of the mid and distal portion of the left anterior descending artery, and cardiomyopathy was considered disproportionate to his coronary artery disease. He had well controlled hypertension and no history of alcohol use. Enzalutamide was discontinued since it was the only preceding event before his onset of heart failure. He was started on guideline-directed medical therapy with Carvedilol and Entresto. The patient declined spironolactone due to concern for side effects. A repeat echocardiogram after three months showed improvement in LV systolic function with an ejection fraction of 45%. Our patient decided to continue ADT therapy alone since he is not symptomatic from his metastatic cancer.

## Discussion

To our knowledge, this is the first case reported that has a direct correlation of enzalutamide to cardiomyopathy. Men treated with ADT have been well shown to have increased cardiovascular risk, and one study predicted a 20% increase in serious cardiovascular morbidity [3]. Enzalutamide is an androgen receptor inhibitor, and it binds to the androgen receptors with a higher affinity than older inhibitors like bicalutamide [4]. A pharmacovigilance study by Eugene B et al. in 2021 found lower cardiac events in patients with Enzalutamide compared to Abiraterone [5], and investigators suggested that enzalutamide may be better suited for patients with known cardiac comorbidities. Our observation is contrary to the fact the patient had excellent tolerance to Abiraterone but not to Enzalutamide. A meta-analysis by Lee HY et al. in 2020 reported a total of 7103 patients from seven RCTs and found Abiraterone had a higher probability of cardiac events than Enzalutamide, but the incidence of hypertension was higher in patients treated with Enzalutamide [6]. Another meta-analysis in 2018 studied a total of 8660 patients and reported a similar result with an increased incidence of hypertension in patients treated with Enzalutamide [7]. These observations concord with the higher incidence of hypertension in patients with Enzalutamide [13% vs. 4%], as observed in the landmark trial comparing enzalutamide with placebo [8]. An interesting observation was made by Moreira R et al., who reported no increased risk of cardiovascular events with Enzalutamide compared to Abiraterone [9]. With regards to specific cardiovascular events, a study in 2020 reported hypertension (10.6%) as the most common cardiovascular event in the Enzalutamide group, followed by ischemic heart disease (1.88%) and atrial fibrillation (0.39%) [10].

## Conclusion

We understand from this review that men treated with ADT have a higher risk for cardiovascular adverse events, and the risk increases with the addition of novel anti-androgen treatments. Overall, Abiraterone appears to have a higher incidence of cardiovascular events, and data are conflicting in patients treated with Enzalutamide. An increase in hypertension has been well documented in patients treated with Enzalutamide. Treating physicians must be prudent in using novel anti-androgen treatments in elderly patients with a detailed discussion about potentially serious side effects of these agents. More prospective research is needed to see if initiating cardioprotective medications will reduce the risk of cardiovascular events in this patient population.

## Data Availability

Not applicable.

## Abbreviations

ADT: Androgen deprivation therapy
GnRH: Gonadotropin releasing hormone
PSA: Prostate specific antigen
LV: Left ventricular
